# Pharmacist-led penicillin allergy interventions and delabeling outcomes: a scoping review

**DOI:** 10.1017/ash.2026.10831

**Published:** 2026-09-23

**Authors:** Ceren Mandacı, Emre Kara, Gökhan Metan

**Affiliations:** 1 Faculty of Pharmacy, Hacettepe University, Turkiye; 2 Department of Clinical Pharmacy, Faculty of Pharmacy, Hacettepe University, Ankara, Turkiye; 3 Department of Infectious Diseases and Clinical Microbiology, Faculty of Medicine, Hacettepe University, Ankara, Turkiye

## Abstract

**Background:**

Penicillin allergy is the most frequently reported drug allergy, yet most labels are inaccurate or no longer clinically relevant. Mislabeling may lead to avoidance of beta-lactam antibiotics, suboptimal antimicrobial therapy, increased healthcare costs, and potential contribution to antimicrobial resistance. Pharmacists increasingly contribute to antimicrobial stewardship programs; however, their role in penicillin allergy evaluation and delabeling has not been comprehensively synthesized.

**Objective:**

To map pharmacist-led or pharmacist-involved interventions aimed at clarifying inappropriate penicillin allergy labels and to describe reported antimicrobial stewardship–related outcomes.

**Methods:**

This scoping review followed the PRISMA extension for scoping reviews (PRISMA-ScR). PubMed, Scopus, and Web of Science were searched for English-language studies evaluating pharmacist-led or pharmacist-involved penicillin allergy interventions among adults receiving systemic antibiotics.

**Results:**

Of 1,269 records screened, 15 studies met the inclusion criteria. Interventions most commonly included structured allergy history assessment and risk stratification, education of healthcare professionals, and facilitation of penicillin skin testing and oral drug provocation testing. Pharmacist involvement was frequently associated with increased beta-lactam prescribing and reduced use of broad-spectrum alternatives. Several studies also reported shorter hospital length of stay and lower healthcare costs compared with usual care.

**Conclusions:**

Pharmacist involvement in penicillin allergy evaluation may help optimize antimicrobial therapy and strengthen antimicrobial stewardship efforts. Integration of pharmacists into allergy assessment workflows may improve the accuracy of allergy documentation and facilitate appropriate beta-lactam use. Further research is needed to evaluate long-term clinical, economic, and implementation outcomes across diverse healthcare settings.

## Introduction

Antibiotics represent a class of medications that are used with high frequency in inpatient and outpatient settings.^1^ Penicillins were the first antibiotics derived from microbial sources and marked the beginning of the antibiotic era.^2^ Since their widespread introduction during World War II, penicillins have saved millions of lives worldwide. This is due to their favorable safety profile, efficacy, and cost-effectiveness across all age groups.^2,3^ Despite these advantages, penicillins are the most frequently reported cause of drug allergy.

Penicillin allergy is often misclassified because many reported reactions reflect childhood intolerance, non-immune-mediated adverse effects, viral exanthems, or family history rather than true hypersensitivity.^4–6^ These inaccurate labels often remain in medical records for years without verification, leading to avoidance of first-line beta-lactam antibiotics and increased reliance on broader-spectrum alternatives.^7^ Such prescribing practices contribute to antimicrobial resistance, increased healthcare costs, and adverse clinical outcomes.^7–9^

Epidemiologic studies indicate that penicillin allergy is the most commonly reported drug allergy worldwide, with prevalence rates ranging from 6% to 25% depending on geographic region and population characteristics.^10,11^ In developed countries, approximately 5%–15% of the general population carries a penicillin allergy label, although only 0.5%–5% of these individuals have a true allergic reaction upon formal evaluation.^12^ Patients with documented penicillin allergy have been shown to have higher mortality, increased hospitalization rates, longer lengths of stay, and higher healthcare expenditures.^8,9,13^

Risk stratification based on clinical history allows appropriate evaluation of patients identified as penicillin allergic. Low-risk patients can undergo direct label removal or drug provocation testing, whereas moderate-risk patients are candidates for penicillin skin testing (PST), and high-risk patients require specialist referral.^14,15^ PST has a high negative predictive value and, when followed by drug challenge, allows safe reintroduction of beta-lactams in most patients.^16,17^ Despite the availability of effective diagnostic strategies, delabeling remains underutilized due to barriers such as limited time, lack of expertise, fear of adverse reactions, and inadequate patient education.^18,19^ Over the past two decades, pharmacist-led antimicrobial stewardship (AMS) interventions have evolved from traditional activities focused on medication review, optimization of antimicrobial therapy, audit and feedback, and prescriber education to encompass a broader range of stewardship interventions. As recognition of the impact of inaccurate penicillin allergy labels on antimicrobial prescribing has grown, these interventions have expanded to include penicillin allergy assessment, risk stratification, diagnostic testing, and delabeling, thereby supporting appropriate beta-lactam use and optimizing antimicrobial prescribing.^20^ In this context, pharmacists are increasingly recognized as key contributors to penicillin allergy management. Their expertise in antimicrobial pharmacotherapy, adverse drug reactions, and beta-lactam cross-reactivity enables them to identify inappropriate allergy labeling, facilitate diagnostic testing, and optimize antimicrobial prescribing.^21,22^

Given the high prevalence and clinical impact of penicillin allergy labeling and the expanding role of pharmacists in AMS, a comprehensive mapping of pharmacist-led interventions is warranted. Therefore, this scoping review aims to evaluate pharmacist-led strategies for the identification, assessment, and management of penicillin allergy labels in community and clinical settings and to examine their impact on antimicrobial use and patient outcomes.

## Methods

### Search strategy

A comprehensive literature search was conducted in PubMed, Scopus, and Web of Science on December 3, 2025, to identify relevant studies published in English. The search strategy was developed in accordance with the PRISMA Extension for Scoping Reviews (PRISMA-ScR) recommendations.^23^ Database-controlled vocabulary and free text keywords were used for five key concepts: “penicillin allergy,” “beta-lactam,” “pharmacist,” “antimicrobial stewardship (AMS),” and “hypersensitivity.” Boolean operators (AND, OR) and truncation were used where appropriate, and the strategy was adapted for each database. For PubMed, the following search string was used (“penicillin allergy” OR “beta-lactam allergy”) AND (pharmacist* OR pharmacy) AND (“antimicrobial stewardship” OR AMS) AND (hypersensitivity* OR allergic reaction*). No date or language restrictions were applied during the search; however, only studies available in English full text were included.

### Study management

All retrieved records were imported into EndNote 20, and duplicates were removed. Deduplicated records were then uploaded to Rayyan® (https://rayyan.ai),^24^ which was used as the platform for title and abstract screening and study management.

### Eligibility criteria

This scoping review focused on studies evaluating pharmacist-led interventions in the identification of penicillin allergy, allergy testing, and the optimization of antimicrobial therapy. Observational studies (prospective or retrospective), pilot studies, and non-comparative designs were reviewed to inform the background and scope of the review; however, eligibility for inclusion in the final synthesis was limited to studies with a comparator or control group, including controlled prepost, quasi-experimental, and randomized or non-randomized study designs. Studies based solely on surveys and those without a pharmacist-led intervention were excluded. Eligible populations included adults (≥18 yr) receiving systemic antibiotics in healthcare settings, whereas studies conducted exclusively in pediatric populations (<18 yr) were excluded. Although this review followed a scoping review methodology, the final synthesis was intentionally restricted to studies with a comparator or control group because the objective of the review was to evaluate the impact of pharmacist-led interventions on AMS and prescribing outcomes.

### Screening and selection

**Figure 1.**
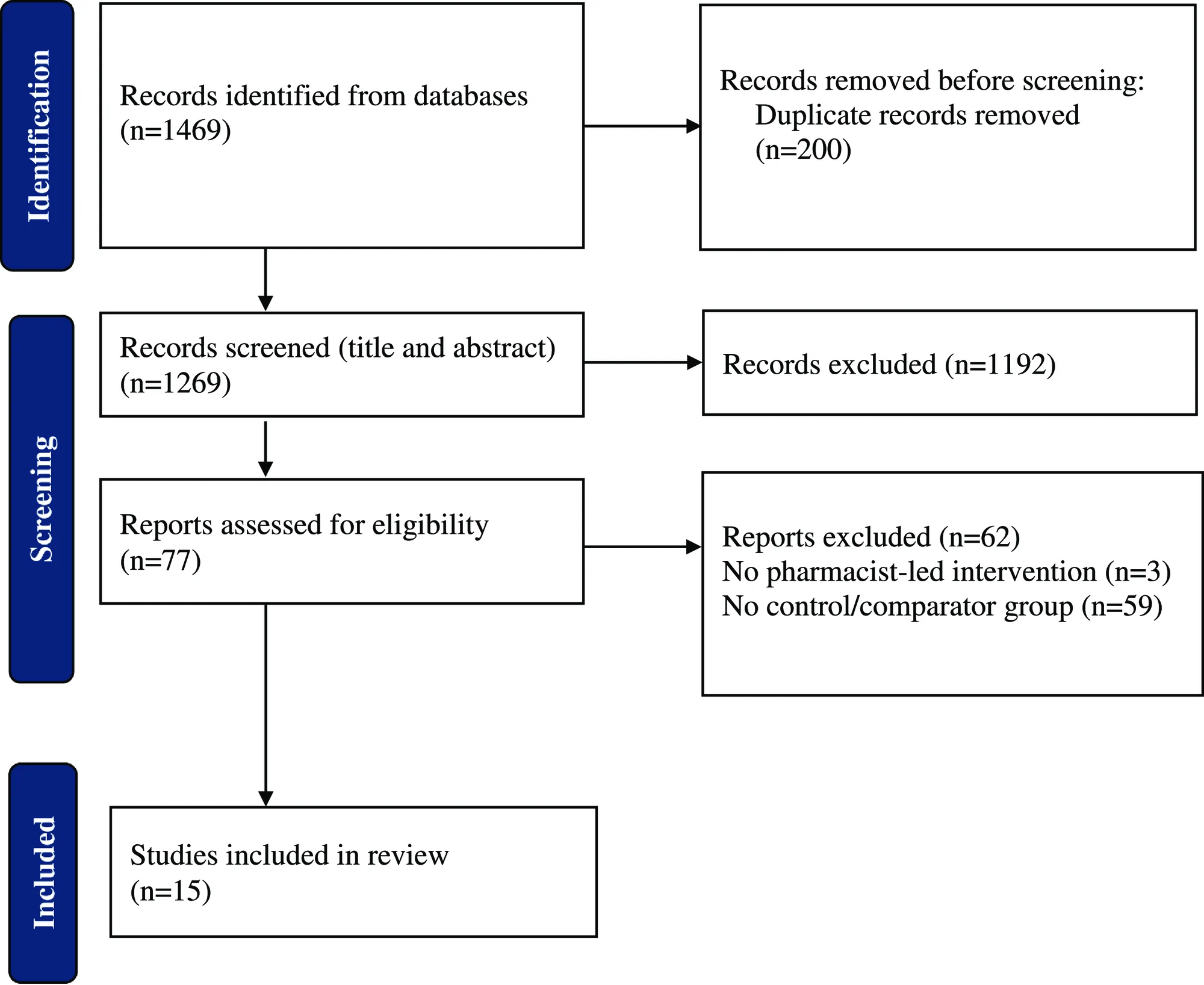
PRISMA-ScR flow diagram of study identification, screening, and inclusion. The figure depicts the study selection process according to PRISMA-ScR guidelines.

Titles and abstracts were independently screened by two reviewers using the Rayyan® platform against predefined eligibility criteria. Rayyan® served solely as the screening platform, and all eligibility decisions were made independently by the two reviewers. Full-text articles were retrieved when abstracts did not provide sufficient information to determine eligibility. Screening decisions were made independently and blinded to the ratings of the other reviewer. Disagreements were resolved by discussion and consensus after full-text re-evaluation. The study selection process is shown in Figure 1.

### Data extraction

Full texts of the included studies were retrieved, and data were extracted using a standardized data abstraction form. Items extracted included study design, setting, patient characteristics and demographics, sample size, pharmacist intervention details, control/comparison group (if applicable), and reported outcomes related to penicillin allergy identification, allergy testing procedures, and antimicrobial optimization. Data extraction was performed by one reviewer and then checked for accuracy and completeness by a second reviewer. As the aim of this scoping review was to map the existing literature rather than to assess methodological quality, no critical appraisal of individual studies was undertaken.

### Synthesis of results

A narrative synthesis approach was used to summarize the findings, with results organized in structured tables according to intervention and outcome domains.

## Results

### Study selection

The literature search identified 1,469 records. After de-duplication and manual review, 1,269 records remained for title and abstract screening, of which 1,192 were excluded for not meeting the inclusion criteria. Seventy-seven full-text reports were assessed for eligibility, and 62 were excluded, primarily due to the absence of a pharmacist-led intervention related to penicillin allergy and/or because outcomes relevant to the intervention were not reported. A total of 15 studies met the inclusion criteria and were included in this scoping review (Figure 1).

### Study characteristics

The characteristics of the included studies are summarized in Table 1. The studies were published between 2016 and 2024 and were predominantly conducted in the United States (n = 13), with additional studies from Australia (n = 1) and Canada (n = 1). Sample sizes (total study populations) ranged from 80 to 2,208 participants. Study populations included adults with documented penicillin or beta-lactam allergy in a variety of clinical settings. The study design, study period, and study setting are detailed in Table 1.

**Table 1.** Characteristics of the studies included in the scoping review Table 1 long description.

Authors, year	Country	Patient population	Number of patients	Study design	Study period	Study setting
Shannon et al., 2016^57^	United States	Hospitalized patients with beta-lactam allergy reported in electronic medical records and prescribed alternative antibiotics	Control: 63 Intervention: 63	Cohort study	October 2014 – April 2015	Public hospital
Swearingen et al., 2016^34^	United States	Adult patients with penicillin allergy record prescribed aztreonam	Control: 117 Intervention: 94	Quasi-experimental study	August 2012–January 2013	Teaching and research hospital
Leis et al., 2017^36^	Canada	Patients in whom beta-lactam treatment was preferred but could not be safely administered due to a beta-lactam allergy label	Control: 246 Intervention: 386	Prospective pre-post comparative intervention study	July 2015–April 2016	Multicenter (1 teaching and research hospital, 2 public hospitals)
Covington et al., 2019^33^	United States	Adults prescribed fluoroquinolones with suspicion of beta-lactam allergy	Control: 43 Intervention: 37	Quasi-experimental study	November 2017–March 2018	Public hospital
Jones et al., 2019^38^	United States	Patients with penicillin allergy who received infectious disease consultation	Control: 100 Intervention: 100	Quasi-experimental study	January 2015–January 2017	2 public hospitals
Modi et al., 2019^39^	United States	Adult hospitalized patients with reported beta-lactam allergy undergoing hematopoietic stem cell transplantation	Control: 136 Intervention: 72	Retrospective cohort study	July 2010–February 2019	University hospital
Campbell et al., 2020^30^	United States	Adult patients who received antibiotic treatment in the emergency department	Control: 255 Intervention: 125	Quasi-experimental study	March 2017–August 2018	Emergency department of a public hospital
Collins et al., 2021^37^	United States	Patients undergoing a surgical operation	Control: 1119 Intervention: 1089	Quasi-experimental study	January 2012–September 2018	Teaching and research hospital
Chua et al., 2021^35^	Australia	Hospitalized patients	Control: 870 Intervention: 355	Cohort study	January–August 2019	Oncology center, teaching and research hospital
Kwiatkowski et al., 2021^32^	United States	Patients with beta-lactam allergy	Control: 50 Intervention: 37	Quasi-experimental study	November 2017–March 2019	University hospital
Quartuccio et al., 2023^29^	United States	Patients in the peripartum period	Control: 96 Intervention: 96	Retrospective study	May 2018–October 2020	Tertiary university hospital and state hospital
Vyas et al., 2023^28^	United States	Hospitalized adult patients receiving antimicrobial therapy with beta-lactam allergy	Control: 184 Intervention: 208	Quasi-experimental study	December 2018–May 2021	Public hospital
Stonerock et al.,2023^58^	United States	Patients with a penicillin allergy label are undergoing surgical procedures	Control: 422 Intervention: 492	Quasi-experimental study	August 2021–March 2022	University hospital
Backus et al., 2024^59^	United States	Adult patients with a penicillin allergy label receiving antibiotics for surgery	Control: 170 Intervention: 170	Cohort study	January 2022–January 2024	Tertiary care public hospital
Hitchcock et al., 2024^31^	United States	Adult patients scheduled for orthopedic surgery	Control: 135 Intervention: 66	Quasi-experimental study	July 2022– February 2023	Tertiary university hospital

### Pharmacist-led interventions and outcomes

Pharmacist-led interventions included structured allergy histories, preoperative allergy clarification, implementation of standardized prescribing guidelines, PST, and oral drug provocation testing (including direct oral challenge in selected low-risk patients), and prescriber education initiatives. Multidisciplinary collaboration involving pharmacists, physicians, and other healthcare professionals was commonly reported.

Structured assessment tools, including the Antibiotic Allergy Assessment Tool, which classifies penicillin allergies into color-coded risk groups and guides de-labeling strategies, have been described in the literature. In addition, validated clinical decision tools such as the PEN-FAST score have been used to support standardized evaluation of reported penicillin allergy.^15,25–27^ Risk stratification was based on structured clinical criteria including reaction type, timing of onset, severity, and time since the reaction. Patients were initially screened to exclude severe non-immediate reactions (eg, Stevens–Johnson syndrome, drug reaction with eosinophilia and systemic symptoms, organ-specific reactions, or hematological complications), as these were considered contraindications for further testing.^14,15^

Based on this assessment, patients were categorized as low, moderate, or high risk. Low-risk histories included isolated non-allergic symptoms, such as gastrointestinal symptoms, pruritus without rash, benign cutaneous reactions, remote (>10 yr) or unknown reactions without features suggestive of IgE-mediated hypersensitivity, or family history alone. Moderate-risk histories included urticaria or other pruritic rashes with features suggestive of IgE-mediated hypersensitivity, without anaphylaxis. High-risk histories included anaphylaxis, positive penicillin skin test results, recurrent reactions, or hypersensitivity to multiple beta-lactam antibiotics.^14^

Management strategies were guided by this risk classification framework. Low-risk patients were considered suitable for direct oral amoxicillin challenge or de-labeling without prior testing. Moderate-risk patients underwent PST prior to oral challenge or, where unavailable, graded oral challenge protocols. High-risk patients were not considered suitable for testing and required specialist evaluation; in selected cases, desensitization procedures were used. In addition, pharmacists contributed by reviewing allergy histories, assessing concomitant medications affecting testing safety, and providing patient counseling and antibiotic stewardship guidance.^14,26,28^

Across studies, pharmacist interventions were generally associated with increased use of guideline-recommended beta-lactam antibiotics. For example, among peripartum patients with penicillin allergy labels, pharmacist-assisted guideline implementation resulted in a 50% increase in first-line beta-lactam use.^29^ In the ED, pharmacist-led allergy evaluations increased prescribing of guideline-recommended antibiotics from 45% to 58% postintervention (*P* = .018).^30^ In surgical populations, pharmacist-led allergy education services were associated with increased cefazolin use and improved allergy documentation.^31,32^

Reductions in the use of broad-spectrum and alternative antibiotics have also been reported. Several studies demonstrated reduced use of vancomycin, fluoroquinolones, and aztreonam after pharmacist intervention.^28,33,34^ Pharmacist-led allergy delabeling strategies, including PST and direct oral challenges, facilitated increased prescribing of narrow-spectrum beta-lactams and reduced reliance on restricted antibiotics.^35,36^

Economic and health care utilization outcomes were evaluated in several studies. Pharmacist interventions have been associated with reduced antibiotic days of therapy, reduced length of hospital stay, and reduced antimicrobial-related costs.^37–39^ In studies reporting safety outcomes, beta-lactam antibiotics were generally well tolerated after pharmacist-assisted allergy evaluation, and serious adverse reactions were uncommon. Overall, the included studies suggest that pharmacist-led penicillin allergy interventions may improve beta-lactam prescribing, reduce the use of alternative broad-spectrum agents, and support AMS and healthcare utilization outcomes in various clinical settings. The objectives, pharmacist contributions, and key findings of the included studies are summarized in Table 2.

**Table 2.** Objectives and outcomes of pharmacist interventions in studies included in the scoping review Table 2 long description.

Authors, year	Study objective	Pharmacist contribution	Outcome of pharmacist intervention
Shannon et al., 2016^57^	To compare the proportion of patients receiving beta-lactam therapy for bacterial infections before and after implementation of a formal allergy evaluation protocol.	Pharmacists performed beta-lactam allergy assessments, updated allergy information in electronic medical records, and provided recommendations to ensure appropriate continuation of antibiotic therapy.	Switching to beta-lactam antibiotics occurred in 57% of intervention patients versus 22% of control patients (*P* = .0019). Of 63 patients with reported beta-lactam allergy, 44% had no documented reaction or an unknown reaction. Most patients tolerated beta-lactam therapy without complications.
Swearingen et al., 2016^34^	To evaluate the effect of a multifaceted antimicrobial stewardship intervention on improving antibiogram-guided antibiotic selection in patients with reported penicillin (PCN) allergy receiving aztreonam.	Pharmacist-led, antibiogram-guided decisions on antibiotic changes, including assessment of allergic reactions, Naranjo adverse drug reaction evaluation, and updating of patient allergy profiles.	In the intervention group, the median duration of aztreonam use decreased from 4 to 2 d (*P* = .0001). Mean aztreonam use per 1,000 patient days decreased from 14.5 to 9.3 d (*P* = 0.0001).
Leis et al., 2017^36^	To evaluate changes in beta-lactam antibiotic use and clinical outcomes following pharmacist-assisted skin testing for beta-lactam allergy.	Pharmacist-administered beta-lactam allergy skin testing was performed in accordance with antimicrobial stewardship practices.	Adjusted analysis showed that the odds of receiving beta-lactam therapy during the intervention period were 4.5 times higher (95% CI: 2.4-8.2; *P* < 0.0001).
Covington et al., 2019^33^	To evaluate the impact of pharmacy-led beta-lactam allergy consultations on fluoroquinolone duration.	Fourth-year pharmacy students conducted allergy consultations under the supervision of senior pharmacists, and pharmacists updated patients’ medical records accordingly.	Fluoroquinolone duration decreased from 3.7 to 2.7 d (*P* = .027). In 49% of patients, therapy was switched to beta-lactam antibiotics without allergic reactions, side effects, or hospitalization.
Jones et al., 2019^38^	To evaluate the impact of PST implementation on antimicrobial prescribing and patient outcomes in a community health system.	Pharmacist-led PST administration, interpretation, and documentation were performed.	Non-penicillin beta-lactam use decreased significantly (*P* < .0001). PST-driven changes in antimicrobial regimens resulted in an average cost savings of $353.03 per patient.
Modi et al., 2019^39^	To evaluate the impact of penicillin allergy skin testing (PAST) in immunocompromised patients undergoing hematopoietic stem cell transplantation (HSCT), focusing on antibiotic use, DOT, and costs of alternative antibiotics.	Pharmacists guided the clinical team in appropriate antibiotic selection based on PAST results, supported integration into the electronic medical record, and promoted the reduction of unnecessary alternative antibiotic use.	Days of therapy (DOT) and cost of alternative antibiotics decreased significantly after implementation (mean 788 vs. 627 days, *P* = .01; mean $24.25 vs. $17.52, *P* = .009). PAST reduced the use of alternative antibiotics, treatment duration, and costs by enabling accurate beta-lactam allergy assessment.
Campbell et al., 2020^30^	To compare prescription rates of guideline-recommended antibiotics (penicillin, cephalosporin) in the emergency department before and after the intervention.	The pharmacist conducted PCN allergy assessments through chart review and patient interviews.	Prescription of guideline-recommended antibiotics increased from 45% (114/255) pre-intervention to 58% (72/125) post-intervention (*P* = .018).
Collins et al., 2021^37^	To ensure the use of beta-lactam antibiotics for surgical prophylaxis in patients with beta-lactam allergy.	A multidisciplinary team, including pharmacists, developed a side-chain cross-reactivity table and optimized allergy assessment procedures.	Use of alternative antibiotics decreased in the intervention cohort compared with the pre-intervention cohort (15.1% vs. 84.9%; *P* < .001). Thirty-day readmissions were also lower in the intervention cohort (6.3% vs. 7.9%; *P* = .035).
Chua et al., 2021^35^	To facilitate the removal of inappropriate allergy labels in adult patients with PCN allergy by direct delabeling or oral drug challenge.	Pharmacists contributed by taking patient histories or reviewing past medical records to assist in delabeling and administering oral drug challenges.	After the intervention, patients with removed allergy labels had a 10.51-fold increase in narrow-spectrum PCN use, a 2.13-fold increase in appropriate antibiotic prescribing, and a 62% reduction in restricted antibiotic prescribing.
Kwiatkowski et al., 2021^32^	To implement and evaluate a pharmacist-led beta-lactam allergy consultation service in the preoperative setting.	Pharmacists conducted preoperative telephone interviews to clarify beta-lactam allergy histories. Based on allergy history and a decision algorithm, first-line antibiotics, alternative agents, or referral for allergy testing were recommended.	All beta-lactam allergy labels were updated following pharmacist consultation. Twenty-three patients were referred for allergy testing; 12 completed testing, and PCN allergy was removed in 9 patients. Cefazolin use increased significantly from 28% to 65% (*P* = .001).
Quartuccio et al., 2023^29^	To evaluate whether updates to institutional guidelines improve the use of first-line antibiotics in peripartum patients with penicillin allergy.	A multidisciplinary team consisting of obstetricians, pharmacists, and infectious disease specialists developed standardized orders to guide antibiotic selection.	The use of first-line penicillins in PCN-allergic patients increased by 50% in various peripartum indications. Allergic reactions to beta-lactams were minimal and manageable.
Stonerock et al., 2023^58^	To evaluate the impact of a pharmacist-led quality improvement initiative aimed at increasing the use of cefazolin as first-line prophylaxis in surgical patients with a PCN allergy label.	Pharmacists stratified patients into high- and low-risk PCN allergy categories and recommended cefazolin for low-risk patients.	Beta-lactam use increased after the intervention (12.6% vs 37.8%, *P* < .001). Use of broad-spectrum antibiotics decreased, including vancomycin (45.3% vs. 29.5%, *P* < .001) and fluoroquinolones (17.3% vs. 8.7%, *P* < .001). The use of two or more preoperative antibiotics also decreased (22.1% vs. 11.4%, *P* < .001).
Vyas et al., 2023^28^	To evaluate changes in complete beta-lactam allergy documentation and antimicrobial DOT before and after implementation of aCAMP.	Pharmacists conducted structured interviews to obtain drug allergy histories. Patients were stratified into low-, moderate-, and high-risk PCN allergy categories or directly delabeled to support appropriate antimicrobial therapy.	Post-intervention, the rate of complete allergy history documentation increased by 19.8% (*P* = .002). Beta-lactam DOT per 1000 patient days increased by 9.34 days (*P* = .009).
Backus et al., 2024^59^	To evaluate the optimal use of antibiotic regimens for surgical prophylaxis in patients with a PCN allergy label before and after a prescriber education intervention.	Pharmacists provided a month-long educational program for anesthesiologists and surgeons, covering PCN allergy literature, the importance of a complete allergy history, cefazolin side chain differences, and indications for cefazolin use or avoidance.	Optimal preoperative antibiotic use increased from 52% (89 patients) before the intervention to 67% (113 patients) after the intervention (*P* = .011), an absolute increase of 15%. The intervention was associated with significant reductions in 90-day all-cause readmissions and acute care length of stay.
Hitchcock et al., 2024^31^	To evaluate the impact of pharmacist-led preoperative beta-lactam allergy counseling on cefazolin prescribing, proportion of patients with clarified allergy status, and associated clinical outcomes in elective orthopedic surgery.	The pharmacist contacted patients one week before surgery to update beta-lactam allergy labels and emailed an intervention algorithm to the orthopedic surgeon with perioperative antibiotic recommendations.	After the intervention, 62% of patients had updated beta-lactam allergy information in the electronic medical record. Patients with removed allergy labels were numerically more likely to receive cefazolin. No differences in clinical outcomes were observed, although documentation of beta-lactam allergy improved.

CI, confidence interval; PCN, penicillin; PST, penicillin skin testing; DOT, days of therapy; HSCT, hematopoietic stem cell transplant; PAST, penicillin allergy skin testing; CAMP, comprehensive allergy management program; DOT, days of therapy; PCN, penicillin.

## Discussion

### Key findings

This scoping review suggests that pharmacist-directed interventions may support improved management of penicillin (and broader beta-lactam) allergy labeling and strengthen AMS efforts. Across the included studies, pharmacist involvement was generally associated with improved antibiotic selection, particularly increased use of guideline-recommended beta-lactams and decreased use of alternative broad-spectrum agents, improved allergy documentation, favorable utilization and cost outcomes, and generally reassuring safety findings. The evidence base consists primarily of hospital-based studies published between 2016 and 2024, most of which were conducted in the United States and utilize observational designs, predominantly cohort and prepost quasi-experimental studies.

### Interpretation in the context of existing literature

The results of this scoping review are consistent with existing evidence that most reported penicillin allergy labels do not reflect current IgE-mediated hypersensitivity.^5,6,12^ Previous studies suggest that IgE-mediated penicillin sensitivity declines over time, with approximately 80% of patients losing sensitivity after 10 years; however, inaccurate labeling may persist due to misclassification of childhood reactions, nonallergic adverse reactions, and incomplete histories as allergy.^5,40^ Consistent with the literature, persistent penicillin allergy labels are associated with avoidance of first-line beta-lactams and increased prescribing of broader-spectrum, less effective, more toxic, and more costly alternatives.^2,3,7^ These patterns have been associated with antimicrobial resistance, *Clostridioides difficile* infection, longer hospital stays, higher readmission rates, and increased mortality.^7–9^

In this context, pharmacist-led approaches identified in the included studies ranged from history-based delabeling in low-risk patients to facilitating PST and drug provocation testing pathways in moderate-risk patients.^14,15,41,42^ Evidence from pharmacist-managed PST services suggests that these models are feasible and safe and are associated with increased beta-lactam use and decreased reliance on alternative agents, even in settings without on-site allergists.^43–45^ Beyond testing services, pharmacists’ expertise in beta-lactam cross-reactivity and side-chain structure can also support safer cephalosporin selection in patients with penicillin allergy labels.^15,43,46,47^ Finally, pharmacist-led education and stewardship efforts may improve multidisciplinary understanding of penicillin allergy assessment and support more appropriate antimicrobial prescribing decisions.^48,49^

### Strengths and limitations

A key strength of this scoping review is the synthesis of pharmacist-led interventions for penicillin allergy, primarily in hospital-based settings. By capturing a wide range of intervention models and stewardship-relevant outcomes, this review provides insight into the feasibility and potential impact of pharmacist involvement in the assessment and management of penicillin allergy in different clinical contexts.

The included studies were heterogeneous in terms of study design, patient populations, intervention components, and outcome measures, limiting direct comparability and precluding quantitative synthesis. Most of the available evidence was derived from observational or prepost study designs, with relatively few randomized controlled trials. In addition, the literature focused predominantly on adult populations and hospital-based services, with limited evidence from pediatric populations and community pharmacy-based interventions.

### Knowledge gaps and future research

Despite growing evidence supporting pharmacist-led penicillin allergy assessment, important knowledge gaps remain. Standardized, evidence-based protocols are needed to better define the role of pharmacists in allergy assessment, delabeling, and diagnostic testing in various healthcare settings. Future studies should evaluate the sustainability and long-term impact of pharmacist-led delabeling programs, including effects on patient safety, healthcare utilization, and antimicrobial resistance-related outcomes and cost-effectiveness across different healthcare systems. Additional research is also needed in pediatric populations and community pharmacy settings where access to allergy specialists may be limited.^13,50^

Despite growing interest in pharmacist-assisted penicillin allergy evaluation, several barriers to implementation remain.^19,51^ Time constraints and competing priorities often limit pharmacists’ ability to obtain a structured allergy history, provide patient counseling, coordinate observation for direct oral challenge/skin testing, and ensure accurate electronic record updates.^19,51,52^ In addition, unclear role definitions and governance (eg, who is responsible for initiating delabeling, authorizing reexposure, and documenting the outcome as “allergy label removal”) and the lack of reliable local protocols may limit pharmacists’ operational authority in practice.^19,53,54^ Finally, gaps in delabeling knowledge/skills, limited experience, and low confidence, as well as fear of triggering reactions or medicolegal concerns, may further limit engagement.^51,53,54^ These findings support implementation strategies that combine clear institutional pathways and role delineation, adequate resources, and competency-based training/credentialing aligned with robust EHR documentation workflows.^19,52,55,56^

This scoping review suggests that pharmacist-led penicillin allergy assessment may support AMS by improving the accuracy and completeness of allergy documentation, facilitating structured assessment pathways, and enabling more appropriate beta-lactam prescribing. Although the evidence base was heterogeneous and largely derived from nonrandomized, observational, and prepost study designs, the findings suggest that pharmacists may play a practical role in multidisciplinary penicillin allergy assessment pathways. Integrating pharmacists into these pathways may represent a pragmatic, evidence-based strategy to mitigate the impact of inaccurate penicillin allergy labeling on antimicrobial prescribing and healthcare utilization. Further research, particularly well-designed randomized controlled trials, is needed to strengthen the evidence base, increase confidence in these findings, and support wider implementation.

## Supporting information

10.1017/ash.2026.10831.sm001 Mandacı et al. supplementary material Mandacı et al. supplementary material

## Data Availability

No new datasets were generated for this study. All data analyzed were derived from previously published studies cited in the manuscript.

## Supplementary material

The supplementary material for this article can be found at https://doi.org/10.1017/ash.2026.10831.

## Table 1. Long description

A table comparing characteristics of studies on beta-lactam allergy. The table has 13 rows and 7 columns. Column headers are Authors, year, Country, Patient population, Number of patients, Study design, Study period, and Study setting. Row 1: Shannon et al., 2016, United States, Hospitalized patients with beta-lactam allergy reported in electronic medical records and prescribed alternative antibiotics, Control: 63 Intervention: 63, Cohort study, October 2014 – April 2015, Public hospital. Row 2: Swearingen et al., 2016, United States, Adult patients with penicillin allergy record prescribed aztreonam, Control: 117 Intervention: 94, Quasi-experimental study, August 2012–January 2013, Teaching and research hospital. Row 3: Leis et al., 2017, Canada, Patients in whom beta-lactam treatment was preferred but could not be safely administered due to a beta-lactam allergy label, Control: 246 Intervention: 386, Prospective pre-post comparative intervention study, July 2015–April 2016, Multicenter (1 teaching and research hospital, 2 public hospitals). Row 4: Covington et al., 2019, United States, Adults prescribed fluoroquinolones with suspicion of beta-lactam allergy, Control: 43 Intervention: 37, Quasi-experimental study, November 2017–March 2018, Public hospital. Row 5: Jones et al., 2019, United States, Patients with penicillin allergy who received infectious disease consultation, Control: 100 Intervention: 100, Quasi-experimental study, January 2015–January 2017, 2 public hospitals. Row 6: Modi et al., 2019, United States, Adult hospitalized patients with reported beta-lactam allergy undergoing hematopoietic stem cell transplantation, Control: 136 Intervention: 72, Retrospective cohort study, July 2010–February 2019, University hospital. Row 7: Campbell et al., 2020, United States, Adult patients who received antibiotic treatment in the emergency department, Control: 255 Intervention: 125, Quasi-experimental study, March 2017–August 2018, Emergency department of a public hospital. Row 8: Collins et al., 2021, United States, Patients undergoing a surgical operation, Control: 1119 Intervention: 1089, Quasi-experimental study, January 2012–September 2018, Teaching and research hospital. Row 9: Chua et al., 2021, Australia, Hospitalized patients, Control: 870 Intervention: 355, Cohort study, January–August 2019, Oncology center, teaching and research hospital. Row 10: Kwiatkowski et al., 2021, United States, Patients with beta-lactam allergy, Control: 50 Intervention: 37, Quasi-experimental study, November 2017–March 2019, University hospital. Row 11: Quartuccio et al., 2023, United States, Patients in the peripartum period, Control: 96 Intervention: 96, Retrospective study, May 2018–October 2020, Tertiary university hospital and state hospital. Row 12: Vyas et al., 2023, United States, Hospitalized adult patients receiving antimicrobial therapy with beta-lactam allergy, Control: 184 Intervention: 208, Quasi-experimental study, December 2018–May 2021, Public hospital. Row 13: Stonerock et al., 2023, United States, Patients with a penicillin allergy label are undergoing surgical procedures, Control: 422 Intervention: 492, Quasi-experimental study, August 2021–March 2022, University hospital. Row 14: Backus et al., 2024, United States, Adult patients with a penicillin allergy label receiving antibiotics for surgery, Control: 170 Intervention: 170, Cohort study, January 2022–January 2024, Tertiary care public hospital. Row 15: Hitchcock et al., 2024, United States, Adult patients scheduled for orthopedic surgery, Control: 135 Intervention: 66, Quasi-experimental study, July 2022–February 2023, Tertiary university hospital.

Navigate back to Table 1..

## Table 2. Long description

The table presents the objectives, pharmacist contributions, and outcomes of pharmacist interventions in studies on penicillin allergy. It has 12 rows and 4 columns. The columns are labeled Authors, year, Study objective, Pharmacist contribution, and Outcome of pharmacist intervention. Each row details a specific study, including the authors and year, the study objective, the specific contributions made by pharmacists, and the outcomes of these interventions. The studies aim to evaluate the impact of pharmacist-led interventions on antibiotic use, patient outcomes, and healthcare utilization. Pharmacists contribute through allergy assessments, guideline adherence, and education. Outcomes include reduced antibiotic use, improved prescribing practices, and better patient outcomes.

Navigate back to Table 2..
